# Skipping on uneven ground: trailing leg adjustments simplify control and enhance robustness

**DOI:** 10.1098/rsos.172114

**Published:** 2018-01-24

**Authors:** Roy Müller, Emanuel Andrada

**Affiliations:** 1 Motionscience, Institute of Sport Sciences, Friedrich Schiller University Jena, Seidelstraße 20, 07749 Jena, Germany; 2 Department of Neurology/ Department of Orthopaedic Surgery, Klinikum Bayreuth GmbH, Hohe Warte 8, 95445 Bayreuth, Germany; 3 Institute of Systematic Zoology and Evolutionary Biology with Phyletic Museum, Friedrich Schiller University Jena, Erbertstraße 1, 07743 Jena, Germany

**Keywords:** human, bipedal locomotion, SLIP, stability, asymmetrical gait

## Abstract

It is known that humans intentionally choose skipping in special situations, e.g. when descending stairs or when moving in environments with lower gravity than on Earth. Although those situations involve uneven locomotion, the dynamics of human skipping on uneven ground have not yet been addressed. To find the reasons that may motivate this gait, we combined experimental data on humans with numerical simulations on a bipedal spring-loaded inverted pendulum model (BSLIP). To drive the model, the following parameters were estimated from nine subjects skipping across a single drop in ground level: leg lengths at touchdown, leg stiffness of both legs, aperture angle between legs, trailing leg angle at touchdown (leg landing first after flight phase), and trailing leg retraction speed. We found that leg adjustments in humans occur mostly in the trailing leg (low to moderate leg retraction during swing phase, reduced trailing leg stiffness, and flatter trailing leg angle at lowered touchdown). When transferring these leg adjustments to the BSLIP model, the capacity of the model to cope with sudden-drop perturbations increased.

## 1. Introduction

**Figure 1.**
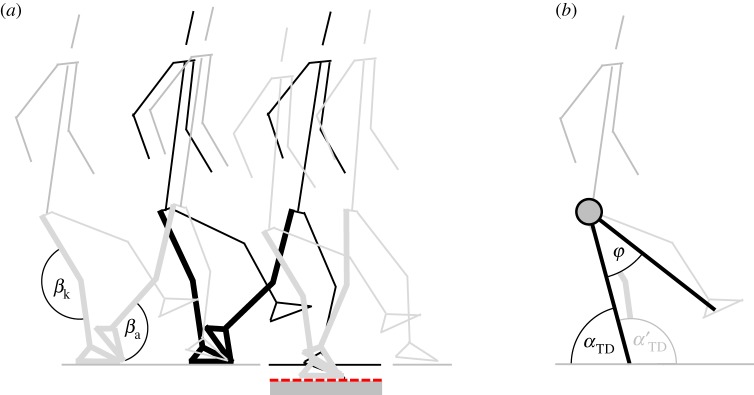
Side view of the instrumented walkway. (*a*) The variable-height force plate was set at two different elevations of 0 cm (black solid line), and −8 cm (red dashed line). Subjects contacted the force plate with the trailing leg (black stick figure) and the leading leg (grey stick figure), respectively. Among others, we calculated the inner angles at the knee (*β*_k_) and ankle joint (*β*_a_) as well as (*b*) the leg angle at touchdown (*α*_TD_) and the aperture angle (*φ*). Please note that several authors would define the leg angle as the complementary angle with respect to the horizontal (*α*’_TD_), meaning that leg retraction during final swing yields a decrease instead of an increase of the leg angle at touchdown.

On Earth, skipping can be observed in humans of any age (e.g. in playing children or in adult humans when descending stairs [1,2], in some birds [3,4], and lemurs [5]). Skipping is an asymmetric^1^ gait (legs kinematics and mechanics differ) that exhibits double support phases and flight phases [1,7]. Hence, the trajectory of the centre of mass shows characteristics shared by both walking and running [1]. Unilateral skipping is a version of this gait in which the same leg (leading leg) is always kept ahead of the other (trailing leg; figure 1).

A recently published study that extracted model parameters from humans skipping over flat ground showed that bipedal spring-loaded inverted pendulum (BSLIP) skipping is self-stable (i.e. the system is able to reject perturbations without any feedback loop) from hopping on the spot to running speeds (authors stopped simulating by human speeds close to 4.3 ms^−1^; [8]). Thus, BSLIP skipping is stable at speeds at which BSLIP walking and BSLIP running are not (BSLIP walking is stable in a narrow corridor around 1 ms^−1^, BSLIP running at speeds higher than 3 ms^−1^; [9]). However, skipping is rather costly for humans and consumes 24% to 30% more metabolic energy and 15% more mechanical energy than running at the same speed [10–12]. Nevertheless, skipping might be energetically beneficial in environments with reduced gravity [13,14]. Thus, skipping is a crucial expansion in our locomotor repertoire when moving on the Moon, and potentially on Mars and could become the primary gait in those conditions [11,13,14].

Whether skipping is used on Earth or on the Moon, perturbations in ground level must be rejected to maintain balance. However, human control strategies that might increase stability of skipping on uneven ground have not yet been addressed. Up to now, strategies used by humans for coping with drops in ground level were analysed for running [15,16] and walking [17,18]. During running on uneven ground, the tolerance to ground disturbances can be enhanced by, e.g. swinging the leg backward before touch down with a constant retraction speed [19,20]. This backward rotational behaviour of the swing leg increases the leg angle at touchdown (figure 1*b*) proportionally to flight duration. As a result of longer flight time and continuous swing leg retraction during running across a sudden drop in ground level, leg angle at touchdown increased in humans and birds [15,21,22]. If swing leg retraction is also used during skipping gaits, the trailing leg (landing first after flight phase) angle at lowered touchdown must increase due to an increased flight phase. That would then be in accordance with a simulation study (mentioned above) which shows that a larger trailing leg angle at touchdown is a very resistant strategy to sudden drops independent of which leg steps into the perturbation [8].

The same model also becomes more robust to sudden perturbations (robustness defined as the maximum perturbation that the system can cope with) when the leading leg (landing last) drops down with a larger leg angle and/or higher stiffness [8]. When compared with the trailing leg, a more retracted leading leg at lowered touchdown can be generated by swing leg retraction but also by a fixed angle between the legs (termed aperture angle) before touchdown. In principle, the two swing leg retraction schemes can increase robustness to sudden perturbations—a mild retraction speed improves walking stability [23,24] and a fixed aperture angle before touchdown improves grounded running [25,26], i.e. running without aerial phases [27]. Nevertheless, the extent to which a swing leg retraction and/or a fixed angle between the legs can be observed during skipping gaits is unknown.

In our investigation, we focused on kinetic and kinematic adjustments during perturbed unilateral skipping across a single drop in ground level. Depending on the leg that steps into the perturbation, we expected that trailing and leading leg adjustments differ. Additionally, based on the experimental findings we extracted possible movement strategies and transferred them to a simple BSLIP template. Finally, we compared and discussed our experimental findings with model predictions.

## 2. Material and methods

### 2.1. Experiments

#### 2.1.1. Measurements

Nine male subjects with no health problems (age: 24.0 ± 1.4 years, body mass: 74.6 ± 6.7 kg, and stature: 179.8 ± 4.7 cm) participated in this study. All subjects were instructed to move using unilateral skipping along a 12 m walkway with one variable-height force plate in its centre (9287BA, Kistler, Winterthur, Switzerland; figure 1). Subjects were allowed to perform several practice trials skipping along the flat track at constant speed. Furthermore, they were free to choose their trailing leg (characterized by the sequence flight-left-right or flight-right-left). After skipping on the unperturbed flat track, the force plate was lowered to an elevation of −8 cm (figure 1). Subjects were visually aware of the single step down and were allowed to perform another two to three practice trials. Under both conditions (even, uneven), the subjects had to accomplish eight trials with touchdown on the force platform. All trials were also recorded by a 3D infrared system (MCU 1000, Qualisys, Gothenburg, Sweden). Reflective markers were placed on the distal head of the first metatarsal bone, on the lateral malleolus, epicondylus lateralis and trochanter major on both sides of the body, as well as on L5 and C7 processus spinosus.

#### 2.1.2. Data processing

Ground reaction force (GRF) was normalized to subject body weight (bw) and a vertical GRF threshold of 0.02 bw was used to determine the instants of touchdown and toe-off. The kinematic data were filtered with a third-order low-pass Butterworth filter (50 Hz cut-off frequency). The virtual leg was defined from trochanter major to a point midway between the distal head of the first metatarsal bone and the lateral malleolus. Stiffness of the leg was calculated as the ratio between the maximum GRF and the maximum leg compression. As leg length is normally non-symmetric relative to touchdown and toe-off events, we used the average leg length at touchdown and toe-off and the minimal leg length during ground contact to calculate the maximum leg compression [8]. Furthermore, we calculated the aperture angle (angle between legs) as well as the inner angles at the knee and ankle joint (figure 1) and estimated the vertical motion of the centre of mass (CoM) by the body segmental analysis method ([28]; not including arm movements). The kinematic analysis focused on the instant of touchdown.

The results were expressed as mean ± s.d. over all subjects and parameters. To compare both kinetic and kinematic parameters, we used repeated-measures ANOVAs (*p* < 0.05; SPSS^®^, Chicago, IL, USA).

### 2.2. Simulations

Skipping was modelled with the BSLIP as introduced in Andrada *et al*. [8]. Below, only the differences with Andrada *et al*. [8] are discussed in detail. The equations of motion of the BSLIP restricted to the sagittal plane are:

$$m\ddot{x}=-k_{T}(l_{0}-l_{T})\;\cos\;\alpha_{T}-k_{L}(l_{0}-l_{L})\;\cos\;\alpha_{L}$$

$$\;\text{and}\;m\ddot{y}=-mg-k_{T}(l_{0}-l_{T})\;\sin\;\alpha_{T}-k_{L}(l_{0}-l_{L})\;\sin\;\alpha_{L},$$

where $\ddot{x}$; $\ddot{y}$ are the accelerations of the CoM, *m* is the body mass, *g* is the gravitational acceleration, *l*_T_ and *l*_L_ are the instantaneous leg lengths during stance, and *α*_T_ and *α*_L_ are the corresponding orientations of the trailing leg (with subscript T) and of the leading leg (with subscript L). In Andrada *et al*. [8], we presented a model with six parameters (*m*, *g*, leg stiffness *k*, trailing leg angle at touchdown *α*_T_, leading leg angle at touchdown *α*_L_, leg length at TD *l*_0_). Based on the experimental observations of the present study, we included in the model the retraction in the trailing leg, the aperture angle between legs, and the asymmetry in the values of the legs stiffness *k*. Thus, the model presented here has seven parameters (*m*, *g,* trailing leg stiffness *k*_T_, leading leg stiffness *k*_L_, trailing leg angle at touchdown *α*_T_ or retraction speed ${\dot{\alpha }}_{T}$, aperture angle between legs *ϕ*_0_ at touchdown, and leg length at touchdown *l*_0_; note that the model needs only six parameters when both trailing and leading leg stiffness are equal). As skipping entails aerial phases, stability considerations can be reduced to a one-dimensional *apex* return-map as described in [8].

The goal of the present simulation study was not the determination of the parameter space in which stable solutions occur. Rather, we were interested in the influence of the movement strategies used by the subjects on the model's behaviour. Therefore, we have first chosen randomly 600 stable periodic solutions from our previous study [8], in which model parameters were similar to those measured in our subjects (75° ≤ *α*_T_ ≤ 85°; 50° ≤ *α*_L_ ≤ 60°; 16 000 Nm^−1^ ≤ *k* ≤ 35 000 Nm^−1^). We then changed the leg angle at touchdown strategy in the leading leg to aperture angle strategy. With such an aperture angle strategy, touchdown of the leading leg occurs when $y=l_{0}\sin(\alpha_{T}-\varphi_{0})$. Afterwards, we checked that the model was still stable (i.e. that the model returned asymptotically to the same periodic solution after a small perturbation; see [8] for details). Note that the eigenvalues provide a measure of how fast a perturbation can be absorbed by the model. However, the eigenvalues do not inform about the size of the perturbation that the model can cope with. The analytical way to measure such robustness is to compute the basin of attraction of a fixed point. We used a rather intuitive and computationally less expensive method closer to our experiments, which is based on sudden drops during forward simulations (called step-down step-up perturbation [8,25,26]). After such a perturbation, the system energy is maintained. SLIP models cannot abolish perturbations with respect to energy.

Following this, we first analysed whether the aperture angle strategy improves the ability of the model to cope with sudden perturbations in relation to the angle of attack strategy. For the representative 600 stable solutions from our previous study, we applied a sudden drop during a forward simulation. Starting at 1 cm, the drop-height was increased up to 10 cm. This procedure was applied to both leg placement strategies. Afterwards, the mean drop-height for every leg placement strategy was computed as an indicator of robustness. For the aperture angle strategy, we identified the simulations at which the model stumbled in the perturbation. For these simulations we performed further forward simulations that included one of the other identified movement strategies (trailing leg retraction, trailing leg more protracted at touchdown, reduced stiffness in the trailing leg) maintaining the drop-height at which the model stumbled. We did this to highlight the single influence of each of these strategies to skipping stability.

The model was implemented in Matlab^®^-Simulink^®^. To integrate the equations of motion, we applied a variable-step algorithm (ode45) with a relative and absolute integrator error tolerance of 1 × 10^−9^ and 1 × 10^−10^.

## 3. Results

### 3.1. Experiments

Four subjects contacted the height-adjustable force plate with the trailing leg and five subjects contacted the force plate with the leading leg. There was no difference in preferred skipping speed on even and uneven ground (table 1). However, when the trailing leg drops down, vertical GRF increased by about 9% from 2.34 bw to 2.54 bw (but the population inference failed to show a difference) and when the leading leg drops down, vertical GRF increased by about 27% from 2.15 bw to 2.73 bw (*p* = 0.001; table 1).

**Table 1.** Kinetic and kinematic parameters. Mean ± s.d. for investigated kinetic and kinematic parameters: skipping speed; contact time (*t*_contact_); maxima of vertical ground reaction force (yGRFmax); vertical positions of the centre of mass at touchdown (yCoM at TD) and toe-off (yCoM at TO); leg angle and leg length at touchdown (TD) and toe-off (TO); aperture angle at TD; retraction and leg stiffness. Significant differences between even und uneven ground (separated for trailing and leading leg) are marked in italic*. *N* denotes number of subjects; *n*, number of total trials. For statistical analysis we used repeated-measures ANOVAs with set-up (even, uneven) as factor.

	trailing leg		leading leg	
	even	uneven	even	uneven
skipping speed (ms*^−^*^1^)	2.9 ± 0.2	2.9 ± 0.2	3.3 ± 0.4	3.1 ± 0.4
*t*_contact_ (s)	0.24 ± 0.03	0.25 ± 0.02	0.27 ± 0.02	0.24 ± 0.02*
yGRFmax (bw)	2.34 ± 0.39	2.54 ± 0.33	2.15 ± 0.16	2.73 ± 0.27*
yCoM at TD (m)	911.2 ± 16.4	855.6 ± 19.0*	840.5 ± 10.6	808.6 ± 16.1*
yCoM at TO (m)	811.6 ± 21.5	776.3 ± 24.8*	930.1 ± 29.7	893.9 ± 41.5*
leg angle at TD (degrees)	82.1 ± 1.4	77.2 ± 1.6*	57.4 ± 2.7	62.3 ± 3.2*
leg angle at TO (degrees)	122.2 ± 0.9	118.4 ± 1.9*	103.1 ± 3.8	101.5 ± 3.7*
leg length at TD (m)	0.99 ± 0.02	0.99 ± 0.03	0.94 ± 0.01	0.95 ± 0.01
leg length at TO (m)	0.97 ± 0.02	0.97 ± 0.01	0.99 ± 0.01	1.00 ± 0.01
aperture angle at TD (degrees)	—	—	49.4 ± 2.5	48.4 ± 2.1
retraction (deg s*^−^*^1^)	4.4 ± 2.0	5.5 ± 2.1	10.3 ± 0.7	11.9 ± 3.0
leg stiffness (Nm*^−^*^1^)	26 026 ± 3618	20 665 ± 4996*	33 442 ± 6817	35 567 ± 6756
*N* (*n*)	4 (22)	4 (25)	5 (26)	5 (29)

Leg adjustments occur mostly in the trailing leg. When the trailing leg drops down, leg stiffness decreased from 26 026 to 20 665 N m^−1^ (*p* = 0.049) and leg angle at touchdown from 82.1° to 77.2° (*p* = 0.011). In addition, we observed no significant kinematic changes in the ankle (even: 93.9 ± 17.0°, lowered: 94.0 ± 20.7°; *p* = 0.979) and knee joint angles (even: 165.2 ± 5.2°, lowered: 162.7 ± 4.0°; *p* = 0.173) at the beginning of the perturbed contact.

**Figure 2.**
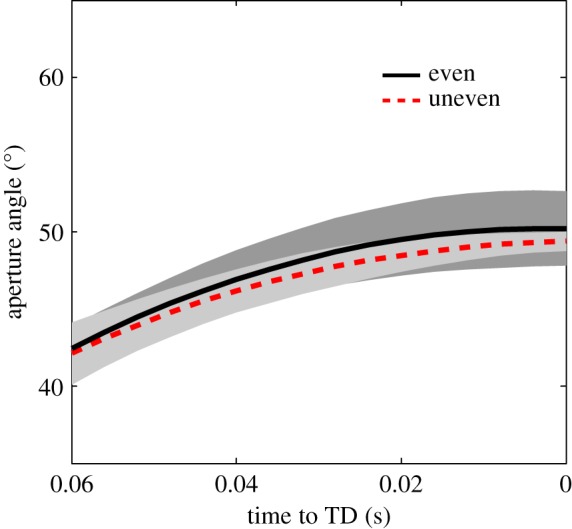
Mean time curves of the aperture angle *φ* from 0.06 s before touchdown (TD) to TD. Black solid line, mean of aperture angle (the leg angle between both trailing (TL) and leading (LL) leg) before even contact; red dashed line, mean of aperture angle before drop step. The standard deviation of aperture angle before even contact is depicted as grey area.

When the leading leg dropped down, leg stiffness remained unchanged (*p* = 0.057) whereas leg angle at touchdown increased significantly from 57.4° to 62.3° (*p* = 0.002; table 1). Additionally, we observed no kinematic changes in the aperture angle (*p* = 0.407; table 1, figure 2) and the ankle (even: 82.5 ± 2.6°, lowered: 80.9 ± 2.2°; *p* = 0.149) and knee joint angles (even: 158.2 ± 5.4°, lowered: 160.3 ± 4.9°; *p* = 0.126) at the beginning of the perturbed contact.

Based on the experimental findings, the following movement strategies were transferred to the numerical BSLIP model:

1. fixed aperture angle (figure 2),
2. low-to-moderate trailing leg retraction during swing phase (table 1),
3. flatter trailing leg angle at touchdown,
4. reduced stiffness in the trailing leg.

### 3.2. Simulations

Mean drop-height values for the angle of attack strategy were 9.7 cm for the trailing leg and 3.2 cm for the leading leg. For the aperture angle strategy, mean drop-height values were 9.5 cm for the trailing leg and 9.6 cm for the leading. Thus, compared to the angle of attack strategy, the aperture angle strategy leads to a larger robustness against sudden-drop perturbations during BSLIP skipping (especially for the leading leg).

**Figure 3.**
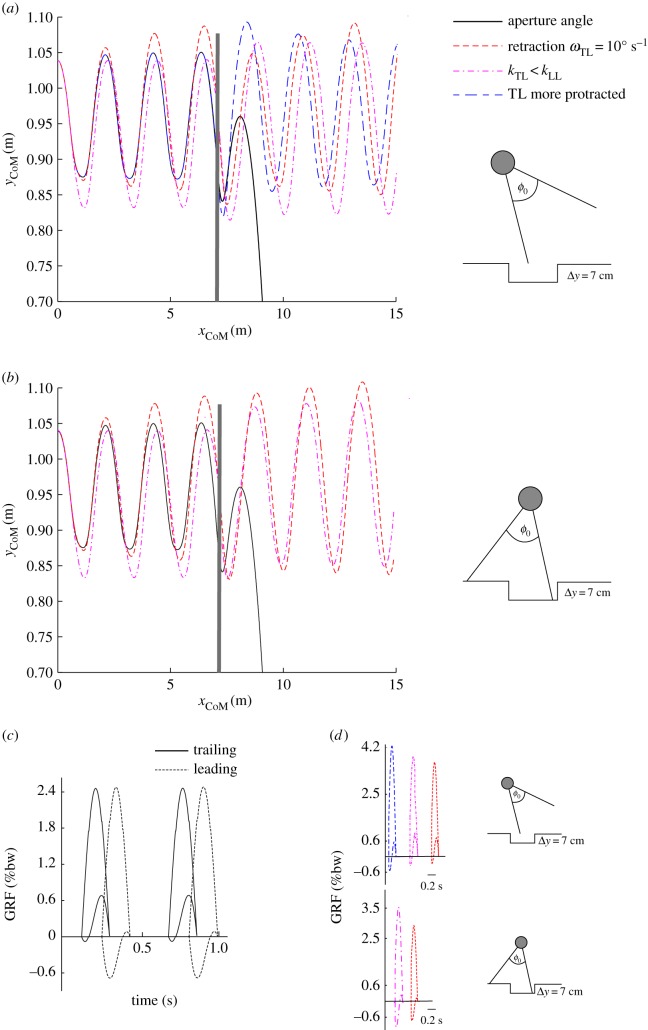
Influence of identified movement strategies on robustness against larger sudden drops. The first simulation was made only with the aperture angle strategy. Every other simulation included only one of the identified movement strategies. (*a*) Trailing leg drops into the perturbation. (*b*) Leading leg drops into the perturbation. (Black solid line) aperture angle; (red dashed line) retraction speed in the trailing leg ${\dot{\alpha }}_{T}=5.7^{\circ }\;{\text{s}}^{-1}$, (magenta dot-dashed line) trailing leg stiffness 30% reduced; (violet dashed line) trailing leg hits in the perturbation with a more protracted angle *α*_T_ = 75°. In (*a*) and (*b*) the grey vertical line indicates the moment at which the leg enters in the perturbation. (*c*) Simulated ground reaction forces during even locomotion (solid line: trailing leg; dashed line: leading leg). (*d*) Simulated ground reaction force at the perturbation. Note that retraction in the trailing leg reduces vertical peak forces. Simulation parameters: *E *= 1445 J; *y*_0_ = 1.079 m; *α*_T_ = 79°; *ϕ*_0_ = 50°; *k* = 20 000 Nm^−1.^

By using the aperture angle strategy, only 10% of the simulations were not able to cope with sudden drops of 10 cm when the trailing leg dropped in the perturbation, and only 8% of the simulations were not able to cope with sudden drops of 10 cm when the leading leg dropped in the perturbation. For those cases, the addition of one of the other identified movement strategies (2, 3 or 4) permitted the model to overcome the perturbation successfully (one example is shown in figure 3*a–b*).

Reduced stiffness in the trailing leg, by extending contact times, prevents (a) that the model engages aerial phases prior to the leading leg touching into the perturbation, and (b) that the model reverts its locomotion direction after stepping in the perturbation. A more protracted trailing leg stepping into the perturbation also permits longer contact times. This helps to generate larger impulses with relative lower leg stiffness. Finally, the rather lower retraction speed also helps the model to cope with sudden drops. Additionally, aperture angle placement strategy leads to the same peak vertical GRFs for the leading and trailing legs including when the stiffness between legs differs (figure 3*c*).

## 4. Discussion

We expected that when humans skip on uneven ground, leg adjustments or leg strategies vary, depending on the leg that steps into the perturbation. Surprisingly, leg adjustments occur mostly or only in the trailing leg. When transferring the identified strategies to the numerical BSLIP model, the capacity of the model to cope with sudden-drop perturbations increased.

**Figure 4.**
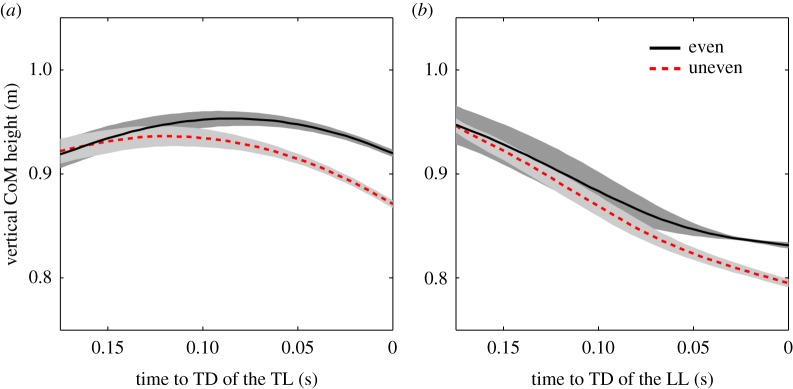
Time course of the centre of mass (CoM) from 0.175 s before touchdown (TD) to TD of one typical subject that contacts the force plate with the trailing leg (*a*) and of one typical subject that contacts the force plate with the leading leg (*b*). (Black line) mean of vertical CoM height before even contact; (red dashed line) mean of vertical CoM height before drop step. The standard deviation of vertical CoM height is depicted as a grey area.

Running stability can be increased by swing leg retraction (e.g. [19,20]). Owing to swing leg retraction, during running on uneven ground the leg angle at touchdown increases proportionally to flight duration [20]. As the flight time depends on ground-level height (the lower the next ground contact, the longer the flight time [29]), a drop in ground level results in a steeper leg angle at touchdown [15,21]. As a result of experimentally observed trailing leg retraction (table 1), we expected a more retracted trailing leg at lowered touchdown (compared to the even contact). Contrary to our expectation, we found that the trailing leg was more protracted and touched the ground with a flatter leg angle (table 1). Despite constant trailing leg retraction (table 1), this result can be (in part) attributed to a flatter and thus, a shorter flight phase prior to the drop step (figure 4) and suggests that the subjects were aware of the perturbation and lowered their centre of mass in preparation of the drop (by about 70% of drop-height, table 1). Nevertheless, since we do not assume that the flight time prior to the drop step is shorter than the flight time to the even contact, it seems that the subjects chose a flatter trailing leg angle at lowered touchdown at will.

Both flatter trailing leg angles at touchdown and decreased trailing leg stiffness during lowered contact (table 1) suggest that humans control and adapt their trailing leg parameters in part actively. In the BSLIP model a more protracted trailing leg permits larger contact times, thereby helping to generate larger impulses necessary to step out of the perturbation. Lower leg stiffness in the trailing leg becomes necessary to prevent that the model reverts its motion direction after stepping in the perturbation and permits flatter aerial phases.

When the leading leg drops into the perturbation, we found steeper leg angles (leg more retracted) at lowered touchdown compared to the even contact (table 1). Such an adaptation can be (in principle) attributed to swing leg retraction or a fixed aperture angle between the legs. By using a fixed aperture angle (table 1), the retraction of the leading leg is coupled with the rotation of the stance (trailing) leg [30]. This coupling may be simply controlled by combining signals from muscle spindles of only a few muscles and an internal kinematic model [30]. Furthermore, the stiffness and length (attributable to unchanged knee and ankle joint angles at touchdown; table 1) of the leading leg did not change between even and uneven conditions. Thus, it seems that humans do not adjust model-related parameters (global control) in their leading leg during unilateral skipping. These findings might indicate a reduction of the control effort. Hence, simplified control, stability and robustness might be the reasons why this gait is used in special situations by humans (e.g. in playing children or in adult humans moving on the Moon's uneven and slippery surface as shown by the astronauts from Apollo missions [1,7,11,13,31]) or in locomotion for other bipeds (e.g. lemurs and birds [3–5]).

For daily human locomotion, skipping is expensive in terms of energetic cost (human skipping consumes 24% –30% more metabolic energy than during running at the same speed [1,10–12]). However, skipping seems to be energetically beneficial in reduced gravity environments. Thus, the selection of skipping in contrast to the emergence of a new gait in hypogravity might indicate that energy consumption triggers changes between gaits, but gait changes occur only between self-stable ones. In few words, self-stability may be a necessary condition for energy minimization. Trade-off between self-stability and energy minimization might exist in facultative bipeds. However, as suggested in Andrada *et al*. [8], the crouched leg configuration observed in small animals might help to decrease the cost of locomotion of skipping. This point and the role of the trunk [32] during skipping are important points that need to be addressed in future studies.

## Footnotes

1 In our paper gait asymmetry should not be understood in the sense of Hildebrand (time-(a) symmetry, [6]), rather that leg movements and mechanics of one side are not exactly repeated on the other side.

## Data accessibility

Kinematic and kinetic data are deposited at https://doi.org/10.6084/m9.figshare.4868336.v1.
